# A Systematic Narrative Review of Stable Staff and Trainers’ Occupational Health and Well-Being in Thoroughbred Horse Racing: An International Perspective

**DOI:** 10.1177/21650799251388471

**Published:** 2025-12-16

**Authors:** Georgia Tobin, SarahJane Cullen, Arthur Dunne, Giles Warrington, Jennifer Pugh, Adrian McGoldrick, Carol Nolan, Cíara Losty

**Affiliations:** 1 Department of Sport and Exercise Science, South East Technological University; 2 School of Health and Human Performance, Dublin City University; 3 Sport and Human Performance Research Centre, University of Limerick; 4 Irish Horse Racing Regulatory Board, The Curragh; 5 Horse Racing Ireland, The Curragh

**Keywords:** injuries, anxiety, depression, workplace health, mental health

## Abstract

**Background:**

Stable staff and trainers are essential to racing yards and horse welfare, yet limited research focuses on this vital workforce that the thoroughbred industry heavily relies on. This systematic narrative review synthesizes existing literature on the occupational health and well-being of stable staff and trainers in the thoroughbred horse racing industry.

**Methods:**

A systematic narrative review was conducted across PubMed, Google Scholar, and Scopus using relevant keywords. Manuscripts reporting occupational or mental health data on stable staff and/or trainers and had the full-text available were synthesized narratively in line with PRISMA guidelines.

**Findings:**

Of 2,456 studies screened, 21 met eligibility criteria. In the past year, 43% of staff experienced an injury, with 18% requiring medical attention. Mental health concerns were prevalent, with 65% of injured staff experiencing anxiety, 60% depression, and up to 80% of UK stable staff and trainers reporting stress linked to financial pressures and limited support. Poor sleep contributed to self-reported daytime dysfunction and psychological distress. Substance misuse was notable, with 34% needing treatment for alcohol and 14% for drug misuse. The staffing crisis was influenced by occupational illnesses, poor working conditions, inexperienced staff, and limited career progression.

**Conclusions/Application to Practice:**

Occupational health challenges highlight the need for interventions to improve mental health support, workplace safety, and working conditions. Industry governing bodies and racing authorities should implement frameworks and standards that ensure safe, supportive, and sustainable work environments. Racing yards should prioritize career progression and educational programs to safeguard the well-being and longevity of this workforce.

## Background

Horse racing is one of the most popular sports in the world, generating billions of revenues annually (Klemm, 2025). The main racing jurisdictions worldwide are Australia, Hong Kong, Ireland, United Kingdom, the UAE, Japan, France, and the United States (The Irish Field, 2024). Central to the success of horse racing is the efficient functioning of racing yards, where the day-to-day care and training of thoroughbred horses takes place.

The structure of a horse racing yard can be best described as a hierarchical pyramid. At the top of the pyramid is the trainer, who serves as the main employer (Juckes et al., 2021). Following the trainer are the stable staff, starting with the assistant trainer and head lad/lass. Next in line are the racing secretaries, senior staff, and traveling head staff. Finally, the exercise/work riders and mucking out staff square off the bottom of the pyramid (Davies et al., 2022).

Trainers play a vital role in the day-to-day operations; they are the employers in the racing yards responsible for the welfare of all employees and horses in their care. Stable staff equally play a crucial role in the running of racing yards and the welfare of thoroughbred horses. Work riders may not conform to the same competitive pressures as jockeys, but they should also be considered athletes, similarly to jockeys, as they exercise multiple thoroughbred horses a day and must also conform to certain physical standards (weight & physically fit) and cope with similar psychological stressors (McConn-Palfreyman et al., 2019). The working environment within thoroughbred horse racing consists of long working hours (more than 9 hours a day), with an increase in weekend work due to the growth in the number of fixtures on the calendar (Juckes et al., 2021). Combined with limited time off and a lack of career progression, the thoroughbred industry is facing an uncertain future. To create a positive working environment, the thoroughbred horse racing industry should satisfy the needs of its employees by ensuring effective working conditions, promoting job satisfaction, and supporting long-term career development.

Mental health and well-being within the workplace have become an increasing concern for employers (Kelloway et al., 2023). The effects of poor mental health can be seen in a lack of focus, increased fatigue, burnout, and withdrawal, yet they extend far beyond the individual, impacting productivity, increasing absenteeism and turnover, and raising the cost of disability leave for companies (Kelloway et al., 2023). A study by Hyland et al. (2022) found that 42.5% of Irish adults met the criteria for a mental health disorder. The most common mental health difficulties employees suffer from include depression, anxiety, and substance use disorders. The workplace itself can have a significant impact on the mental health of its employees. Occupational stress from work can manifest in both physical and psychological consequences (Kelloway et al., 2023). Within the workplace, employers have noted that it is challenging to gauge the mental health of their employees due to the stigma that can be present in the workplace (Wu et al., 2021). Research on employee mental health is often generalized, leading to the neglect of certain worker groups and a disregard for their unique working environments (Kelloway et al., 2023). Limited research is available on the unique working environments in thoroughbred horse racing, with the studies that are available detailing the long working-hours, lack of time-off, and extensive overtime that stable staff and trainers are managing (Juckes et al., 2021; McConn-Palfreyman et al., 2019; Speed & Andersen, 2008).

The thoroughbred horse racing industry has faced some challenges in recent years with a call for racing to demonstrate their high standards of horse welfare and upskill staff and trainers to meet those standards (Davies et al., 2021). The staffing crisis has received media attention due to the large numbers of staff leaving the thoroughbred industry (24% per annum (Public Perspectives Ltd, 2021)). Studies have found that a lack of recognition and respect, alongside the working conditions, inexperienced staff, and career progression are contributing to a substantial proportion of employees considering leaving the industry (Bush et al., 2018; Juckes et al., 2021). Staff retention is therefore affected with the staff-to-horse ratio diminishing in yards, additionally with trainers not in a position financially to employ more stable staff to care for the number of horses they have. Stable staff and trainers also tend to prioritize the horse’s health and welfare above their own (Davies et al., 2021) leading to a workforce that dismisses their own well-being. As a result, this can lead to higher levels of personal, physical, and mental stress, increasing the risk of occupational injuries and consequently impacting the number of employees. Hence, the objective of this narrative review was to synthesize the existing literature concerning the occupational health and well-being of stable staff and trainers. Across different jurisdictions, terms such as racing grooms, back-stretch workers, horse care workers, stable lad/lass, and hot walkers are commonly used interchangeably to refer to what this review defines as stable staff. Work riders or exercise riders are typically considered part of stable staff, although some studies report them separately in their findings, this distinction is noted where relevant. This review aims to examine the current evidence on the occupational health and well-being of stable staff and trainers in thoroughbred horse racing. Specifically, it seeks to address: (1) what factors influence the occupational health and well-being of stable staff and trainers? and (2) what is currently understood about their working conditions and related outcomes?

## Methods

### Search Strategy

A systematic literature search was conducted to identify relevant studies on the occupational health and well-being of stable staff and trainers within the thoroughbred horse racing industry. A literature search was conducted from February 2024 to June 2024 on the following databases PubMed, Google Scholar, and Scopus, covering publications from database inception to June 2024. Additionally grey literature was identified through manual screening of reference lists, relevant industry publications, and non-peer-reviewed reports with primary data or internal research commissioned by racing organizations or welfare bodies. The search strategy combined population terms (e.g., stable staff, racehorse groom) with health-related terms (e.g., mental health, occupational health), using Boolean operators (“AND”/“OR”).

### Eligibility Criteria

To be included in this systematic narrative review articles must have: (1) a sample wholly or partially inclusive of stable staff and/or trainers working in the horse racing industry; (2) included quantitative or qualitative data on the occupational health or mental health of stable staff or trainers, in line with the aims of this study; and (3) have the full text available. Studies were excluded from the review if they met the following criteria: (1) book chapters, conference abstracts, narrative or systematic reviews, or opinion pieces; (2) not written in English.

### Study Selection

All search results were imported into Zotero Reference Manager, where duplicates were identified and removed. Screening for eligibility was conducted by one researcher (G.T), who reviewed all titles and abstracts, followed by full-text assessment. Final selections for inclusion were then presented to and discussed with co-authors (C.L & S.J.C) to ensure agreement and transparency in the selection process. The review was carried out following the recommendations of the Preferred Reporting Items for Systematic Reviews and Meta-Analyses (PRISMA). Articles were reviewed for methodological clarity, transparency of data collection, and relevance to the review.

### Data Extraction and Synthesis

A structured data extraction process was undertaken to ensure consistency and rigor across all included studies. Key data were manually extracted and recorded in an Excel spreadsheet. Extracted data covered study characteristics such as author(s), year of publication, country, study design, sample size, participant population, and key findings. A summary of study characteristics can be found in Table 1. A narrative synthesis approach was employed to analyze extracted data. Studies were organized thematically based on recurring topics including occupational injuries, mental health, substance misuse, sleep quality, and staffing crisis. If a study covered multiple variables, it was categorized according to the most prominent theme. This method of categorization ensures clarity and consistency in identifying the primary focus of each study. This thematic framework guided the interpretation of findings, highlighting common patters and differences across study design, populations, and geographic contexts.

**Table 1.** Summary of Research Examining the Occupational Health and Well-Being of Stable Staff and Trainers

Author & year	Country	Design	Participants (m/f)*	Population	Variable measured	Data collection	Summary
*Occupational injuries*							
Castañeda et al. (2010)	USA	Quantitative survey	*N* = 84 (70/14)	Stable staff & trainers	Medical history & health	Self-made survey	Primary health concerns were muscular/joint/ligament injury or pain, followed by gastrointestinal pain. 29.6% (*n* = 21) had a serious accident or injury in the past 5 years, involving traumatic head injuries.
Cowley et al. (2007)	Australia	Observational	*N* = 985	Stable staff & trainers	Injury statistics	Retrospective analyses	289 claims were made by Racing Victoria Limited (RVL) employees. Non-RVL employees made 696 claims, which consists of stable staff and work riders (71% of claims).
Davies et al. (2022)	UK	Cross-sectional survey	*N* = 198 (37/155)	Stable staff	Injury statistics	Self-made survey based on previous research	310 staff reported injuries. High self-reported incidents of pain, musculoskeletal injuries, and concussion. 75% of staff would take time off for a fracture but only 48% for a concussion.
Davies et al. (2023)	UK	Cross-sectional survey	*N* = 175 (29/140)	Stable staff	Injuries and mental health	HADS	711 injuries were reported. 65% reported mild, moderate, or severe anxiety scores. 59% reported depression scores above the threshold.
Filby et al. (2012)	UK	Cross-sectional survey	*N* = 2,293 (326/1,967)	Stable staff & trainers	Injury statistics	RIASS	53% of accidents were riding accidents. 47% ground-level accidents. 261 accidents resulted in absence from work, with a total of 7,431 sick days taken in the year. Substantial number of walking wounded staff as 60% of accidents resulted in no sickness days.
Iba et al. (2001)	Japan	Observational	*N* = 581 (424/157)	Stable staff	Injury statistics	ISS	637 horse-related injuries were treated in the hospital. Kicks were the most common injury which occurred whilst handling horses. Falls were the second most common injury.
J. E. Swanberg et al. (2016)	USA	Quantitative survey	*N* = 225 (193/32)	Stable staff	Injury statistics	NAWS BLS OASHI (adapted)	43% experienced an injury in the past year. 18% receiving medical attention. The task in which stable staff were injured the most was taking horses in and out of fields. Most common injury locations were wrists & hands (26%), feet (19%), and back (12%).
J. Swanberg et al. (2017)	USA	Cross-sectional survey	*N* = 225 (193/32)	Stable staff	Musculoskeletal discomfort & work factors	PSCS OSHBD	85% of participants experienced MSD. Elevated MSD was associated with longer employment, longer working hours, and poor safety climate.
*Mental health*							
Byrd (2014)	USA	Quantitative	*N* = 73 (46/21)	Stable staff & trainers	Health insurance and impact on health	SF12v2	Non-English speakers scored lower for anxiety and depression. Work riders scored lower on physical and mental health measures.
Kiesler et al. (2013)	USA	Mixed methods	*N* = 50 (42/8)	Stable staff	Social determinants of health	Self-made survey photovoice	71% of Latino respondents use the health clinic at the racetrack compared to 64% of non-Latinos. 30% had been told by a GP that they had depression. Worker’s health and safety was at risk due to high drinking and drug use. Poor living conditions were reported.
Kim et al. (2019)	Korea	Cross-sectional survey	*N* = 207 (207/0)	Stable staff	Occupational stress and depression	KOSS CES-D	Senior assistant trainers had the highest occupational stress in the physical working environment whilst stable staff had the highest occupational stress in the job autonomy factor. 34% of participants met the requirements for depression.
King et al. (2021)	Ireland	Cross-sectional survey	*N* = 124 (100/22)	Trainers	Mental health, alcohol use, social support, help-seeking, and career satisfaction	K10 CES-D GAD AUDIT-C MSPSS CSS	45% of trainers met the criteria for at least one CMD, 32% for at least two and 21% for at least three. Only 21% had accessed mental health support.
McConn-Palfreyman et al. (2019)	UK	Mixed methods	*N* = 131 (60/13)	Stable staff & trainers	Mental health and working patterns	Focus groups & unvalidated survey	Nearly 80% of stable staff and trainers are experiencing stress, anxiety, and depression. 76% of trainers expressed that they do not receive adequate support for mental health within the industry, with 65% for stable staff. 53% of trainers expressed being strong in front of colleagues or peers as the main reason influencing not seeking help with 52% of stable staff expressing the same.
Speed and Andersen (2008)	Australia	Mixed methods	*N* = 693 (373/314)	Stable staff & trainers	Working practices & support services.	Survey & written statements with open-ended questions	1/4 of trainers experienced harassment or bullying in the workplace. Gambling and alcohol use were also problematic for several trainers. Stable staff were found to be struggling financially, working long days and overtime, suffering from harassment and bullying, felt fatigued, stressed, and had poor sleep quality.
*Substance misuse*							
Schefstad and Tiegel (1997)	USA	Qualitative	*N* = 120 (120/0)	Stable staff	Addiction severity	ASI	20% reported illicit drug use within the past 30 days. 34% needed treatment for alcohol use. 14% needed treatment for drug use. 14% reported experiencing psychological or emotional problems that interfered with their ability to work and their relationships.
Schefstad et al. (1999)	USA	Case study	*N* = 700	Stable staff	Evaluation of treatment services for addiction.	Observational	Case study on the efficacy of a program designed to provide services for backstretch stable staff with addiction and mental health difficulties.
*Sleep*							
Bullock et al. (2019)	Australia	Cross-sectional survey	*N* = 358 (229/129)	Stable staff & trainers	Psychological distress and sleep quality	K10 PSQI	Trainers reported higher psychological distress, anxiety, and depression compared to non-trainers. 62% of trainers scored above the cut-off for poor sleep.
Farmer et al. (2017)	Australia	Cross-sectional survey	*N* = 358 (229/129)	Stable staff & trainers	Psychological distress and sleep quality	K10 PSQI	Trainers had higher levels of psychological distress lower sleep quality, daytime dysfunction and high levels of depression compared to the non-trainers regardless of demographic characteristics.
*The staffing crisis*							
Bush et al. (2018)	USA	Quantitative	*N* = 225 (193/32)	Stable staff	Health insurance, physical and mental health, working conditions.	NAWS SF12 CES-D JUSTA (adapted)	32% of stable staff had suffered a work-related illness in the past year. 44% of stable staff reported going to work despite being ill, due to financial pressure.
Butler et al. (2019)	UK	Qualitative	*N* = 42 (20/22)	Stable staff & trainers	Challenges to racehorse welfare.	Focus groups	Poor employee relations due to lack of recognition, communication, and respect affect employee attitudes, behavior, and staff retention which compromises horse welfare.
Juckes et al. (2021)	UK	Qualitative	*N* = 30 (14/16)	Stable staff & trainers	Staffing crisis.	Focus groups	67% of the participants had considered leaving the industry. Four themes emerged: skills, work ethics, job conditions, and media. High workloads, poor work-life balance, and lack of training are negatively influencing retention.

*Note.* Stable staff are inclusive of assistant trainers, racing grooms, hot walkers, horse care workers, backstretch staff, stable lad/lass, traveling staff, work/exercise riders, and secretaries. m = male, f = female (where applicable). HADS = Hospital Anxiety and Depression Scale; RIASS = Racing Injury Accident Statistical Survey; ISS = Injury Severity Score; BLS = Bureau of Labor Statistics; OASHI = Onsite Aquaculture Safety & Health Interview; PSCS = Perceived Safety Climate Scale; OSHBD = Occupational Safety & Health Body Discomfort Interview Guide; KOSS = Korean Occupational Stress Scale; CES-D = Centre for Epidemiologic Studies Depression Scale; K10 = Kessler Psychological Distress Scale; GAD = Generalized Anxiety Disorder; AUDIT-C = Alcohol Use Disorders Identification Test; MSPSS = Multidimensional Scale of Perceived Social Support; CSS = Career Satisfaction Scale; ASI = Addiction Severity Index; PSQI = Pittsburgh Sleep Quality Index; NAWS = National Agricultural Workers Survey; SF-12 = Short Form Health Survey; JUSTA = Justice and Health for Latino Poultry Workers Study Survey.

## Results

**Figure 1.**
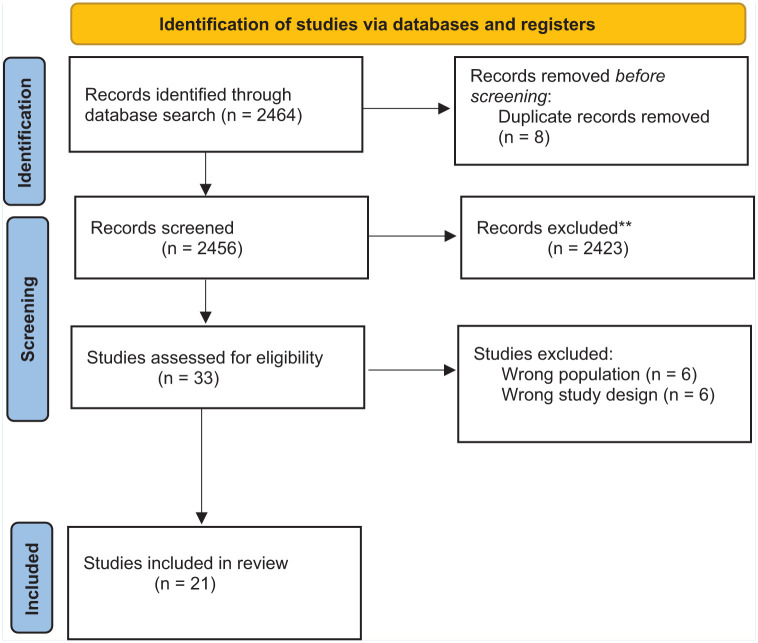
PRISMA flow diagram of study selection

The study selection process is illustrated in Figure 1. The database search identified 2,464 publications, including eight duplicates. After screening 2,456 titles and abstracts, 2,434 were excluded for not meeting the inclusion criteria. Of the 33 full-text articles assessed for eligibility, six were excluded for focusing on the wrong population and another six for using the wrong study design. Ultimately, 21 studies met the inclusion criteria for the systematic narrative review.

### Study Characteristics

The included studies were published from 1997 to 2023. The studies included stable staff and trainers from the USA (*n* = 8), UK (*n* = 6), Australia (*n* = 4), Ireland (*n* = 1), Korea (*n* = 1), and Japan (*n* = 1). Most of the studies used a cross-sectional design (*n* = 8), with the remainder using a quantitative design (*n* = 4), a qualitative design (*n* = 3), mixed methods (*n* = 3), an observational design (*n* = 2), and a case study (*n* = 1). Studies used a mix of stable staff and trainers (*n* = 10), stable staff (*n* = 10), and solely trainers (*n* = 1). See Table 1 for full characteristics of the included studies. Across the 21 studies, several occupational health issues were revealed. These included occupational injuries (*n* = 8), mental health (*n* = 6), substance misuse (*n* = 2), sleep (*n* = 2), and the staffing crisis (*n* = 3).

### Occupational Injuries

Injuries among stable staff and trainers in the thoroughbred industry represent a significant occupational hazard, with studies consistently identifying musculoskeletal injuries and horse-related trauma as primary concerns (Cowley et al., 2007; Iba et al., 2001; J. E. Swanberg et al., 2016). Across diverse geographical contexts, common injuries include kicks, falls, and trampling, predominantly occurring during ground-level activities such as grooming and handling horses (Filby et al., 2012; J. E. Swanberg et al., 2016). In Japan, 39% of stable staff injuries were caused by horse kicks alone (Iba et al., 2001), while in Australia, 70% of compensation claims were due to horse interaction, with stable staff and work riders accounting for 71% of all claims (Cowley et al., 2007). In one recent study, Davies et al. (2022) found that among stable staff, 1,164 injuries were reported in a single year, yet only 11.9% of participants reported no injuries at all.

Workplace safety is further influenced by the interplay between socioeconomic, demographic, and environmental factors. Castañeda et al. (2010), investigated Latino migrant stable staff in the United States, and found that nearly 30% had experienced a serious injury in the last 5 years, with musculoskeletal pain being the most common health concern. Their findings link physical risk to stress, poor living conditions and diet, raising concerns about the compounded vulnerabilities experienced by migrant stable staff. Similar issues were echoed in J. E. Swanberg et al. (2016) and J. Swanberg et al. (2017) studies, which revealed that 43% of participants had sustained an injury in the past year, with musculoskeletal discomfort reported by 85%. Factors such as longer working hours, work experience, and poor safety climate were all significantly associated with discomfort, indicating that discomfort is not only a result of isolated incidents but is tied to broader working conditions and organizational culture.

While much of the literature focuses on physical harm, Davies et al. (2023) explored the psychological impact of injuries for the first time. Of the injured participants, 65% experienced anxiety and 60% met the threshold for depression. Injured staff were also likely to use drugs and alcohol to continue working normal hours. Specifically, stable staff and trainers increased their use of non-steroidal anti-inflammatory drugs (NSAIDs) when injured to continue working which in turn showed increased levels of anxiety and depression (Davies et al., 2023).

### Mental Health

Six studies focused on the mental health and well-being of stable staff and/or trainers in the thoroughbred industry, consistently identifying high levels of mental health concerns linked to structural features of the horseracing industry. Across different national contexts common themes of chronic stress, poor work-life balance, low wages, stigma around help-seeking, and limited mental health support appeared.

An initial study by Speed and Andersen (2008) highlighted the demanding nature of the industry, with 57% of trainers reporting fatigue and 36% reporting stress, with many not having the luxury of annual leave or a sick day. Stable staff echoed these concerns, citing long working hours, poor pay, and pressure not to take time off, leaving them feeling fatigued, and stressed leading to poor sleeping habits. Both studies found evidence of harassment and bullying in the workplace, specifically female work riders who were subject to verbal, sexual, and emotional bullying. Despite this, only 38% of stable staff reported harassment or bullying (Speed & Andersen, 2008). Poor working conditions were also shown to intersect with socioeconomic vulnerability, especially among migrant or minority staff. Kiesler et al. (2013) and Byrd (2014) both explored mental health among Latino backstretch staff in the U.S., identifying the impact of language barriers, housing instability, and precarious work on mental health difficulties. While Byrd (2014) found some positive comparisons to the general population, these findings are limited by small samples and non-comparable measures, and therefore difficult to substantiate.

In the UK, McConn-Palfreyman et al. (2019) found that 75% to 80% of both trainers and stable staff reported experiencing stress, anxiety, or depression with financial pressures, lack of days off, and inadequate support frequently cited as impactful factors. The study also found significant barriers to accessing mental health services, including stigma, cultural norms of stoicism, and fears of appearing weak. Job insecurity and dissatisfaction were explored by Kim et al. (2019) in South Korea, where increased suicide rates prompted an investigation into the stress levels of stable staff. Over one third met the threshold for high-risk depression due to job demand, organizational systems, and inappropriate compensation. King et al. (2021) provided the most focused investigation into trainers’ mental health using validated measures. Findings revealed that 45% of participants met the criteria for a common mental disorder (CMD), yet only 21% had accessed mental health support. Financial difficulties (64%), career dissatisfaction (28%), and low social support all contributed to the probability of an increase in psychological distress, depression, and anxiety (King et al., 2021).

### Substance Misuse

Only two studies directly focused on the use of alcohol and substances within the thoroughbred horse racing industry. Schefstad and Tiegel (1997) reported that 20% of stable staff used illicit drugs in the previous 30 days, 34% needed treatment for alcohol misuse and 14% for drug misuse. The connection between horse-related injuries and drug misuse was acknowledged but not investigated, yet, participants did report experiencing depression and anxiety, with 14% of participants experiencing further psychological distress (Schefstad & Tiegel, 1997). Schefstad et al. (1999) followed on from their previous research with a case study exploring the efficacy of a program designed to provide services to this population. The program provided diagnostic, evaluation, and therapy services to over 700 stable staff. Even though the study summarized the assistance programs, it did not illustrate the participants within the study nor which services were utilized.

A small number of the other studies collected limited data on alcohol and drug use whilst investigating other primary variables such as injury rates or mental health. Davies et al. (2023) found that stable staff who were injured used alcohol as a coping mechanism. King et al. (2021) measured alcohol use with the AUDIT-C scale and found that 38% of trainers had adverse alcohol use, whereas McConn-Palfreyman et al. (2019) found that only 9% of both trainers and stable staff had problems related to alcohol use. Speed and Andersen (2008) examined alcohol and gambling misuse, where 2% to 4% of trainers and 5% of stable staff had difficulties and 8% of stable staff had or continue to have drug misuse.

### Sleep

Two studies examined sleep quality and the psychological effects among stable staff and trainers in thoroughbred horse racing. Farmer et al. (2017) reported that trainers experienced significantly higher psychological distress and poorer sleep quality than both non-trainers and the general population, which was associated with increased self-reported daytime dysfunction (alertness), and in turn, elevated levels of anxiety and depression (Farmer et al., 2017). Bullock et al. (2019) similarly found elevated psychological distress and poor sleep quality among trainers.

### The Staffing Crisis

Three studies examined the factors affecting workforce sustainability. Bush et al. (2018) reported that 32% of Latino stable staff experienced a work-related illness in the past year in which they called in sick, while 44% of participants attended work even though they were sick. Presenteeism was linked to childcare responsibilities, poor general health, and work stress. Butler et al. (2019) revealed how staffing shortages compromised horse welfare through reduced facility standards, compounded by poor communication and a lack of respect from employers, which contributed to a high turnover and low staff-to-horse ratios. Furthermore, several trainers are not in a position financially to employ more stable staff to care for the number of horses they have. Likewise, the stable staff that they are able to employ often lack the required knowledge, which can negatively impact horse welfare and increase the risk of injuries. Juckes et al. (2021) further emphasized the previous points by finding that 67% of stable staff and trainers had considered leaving the industry. Four high-order themes emerged from the focus groups, citing inadequate training, poor working conditions, and limited career progression as central to the crisis.

## Discussion

The findings of this review demonstrate that stable staff and trainers in the thoroughbred horse racing industry face complex and interconnected occupational health challenges. Physical injuries, particularly musculoskeletal injuries and horse-related trauma, are consistently reported among the studies (Cowley et al., 2007; Davies et al., 2022; Filby et al., 2012; Iba et al., 2001; J. E. Swanberg et al., 2016). However, there is a culture of underreporting, which reflects a workplace that normalizes injuries as part of the job and discourages formal reporting (Cowley et al., 2007; Davies et al., 2022; Iba et al., 2001). Despite the high injury count across the studies, no clear associations were found between injury rates and working hours, job role or experience, suggesting that injuries are not merely the result of individual risk factors but may be embedded in the broader working culture. The study also identified a reluctance to formally report or manage injuries, with participants instead relying on over-the-counter medication or modifying tasks to continue working. These findings point to the limited organizational structures in place to support injury recovery, as well as the potential health consequences of self-managed injuries. Davies et al. (2023) study contributed to the knowledge field of stable staff and trainer’s anxiety and depression following injury, it is worth noting that self-reporting has limitations. Social drug use, alcohol, injuries, and betting can be biased and underreporting of such behaviors may be present. Of note was the lack of knowledge of anxiety and depression levels before injury, therefore this study was unable to imply cause-and-effect of injuries with depression and anxiety (Davies et al., 2023). The data underscores the importance of considering injury as both a physical and mental health risk, particularly in environments where help-seeking may be discouraged.

Overall, the included studies consistently show a high prevalence of injuries among stable staff and trainers, along with a cultural normalization of injuries, shaped by structural, industry, and occupational factors. Several studies adopt descriptive or retrospective designs, which, while valuable in capturing patterns, limit the ability to draw conclusions about causes or evaluate the effectiveness of interventions. Studies have not considered how human and industry factors can contribute to injury risk. In particular, organizational responses, injury prevention strategies, and reporting systems remain underexplored areas in the literature, suggesting a pressing need for future research to address these critical gaps.

Mental health difficulties are prevalent across multiple national contexts, with stress, anxiety, and depression commonly reported among stable staff and trainers (Byrd, 2014; Kiesler et al., 2013; King et al., 2021; McConn-Palfreyman et al., 2019; Speed & Andersen, 2008). Structural and organizational pressures, including long working hours, low pay, job insecurity, limited career progression, and inadequate mental health support, exacerbate psychological distress (King et al., 2021; McConn-Palfreyman et al., 2019). Workplace bullying and harassment can have detrimental effects on employees, leading to direct associations with alcohol misuse, psychological distress, and reduced quality of work life (Rospenda et al., 2023; Zulkarnain et al., 2023). This points to a culture of silence around harassment that has implications for mental health. Stigma is a predominant factor in mental health help-seeking and a common theme in research (Torales et al., 2023), and interventions to improve mental health literacy and create more supportive work environments could reduce negative attitudes and discrimination toward mental health (Ramírez-Vielma et al., 2023). Socioeconomic vulnerabilities, particularly among migrant or minority staff, intersect with occupational stressors. Language barriers, housing instability, and precarious work amplify risk, suggesting that mental health challenges are systemic rather than incidental outcomes (Byrd, 2014; Kiesler et al., 2013). Across these studies, methodological limitations, including self-reported data and low sample sizes constrain generalizability. However, the convergence of findings across regions and roles strongly suggests that mental health difficulties among stable staff and trainers are not isolated or incidental but reflect engrained industry structures that demand policy and cultural change. There remains a need for intervention-based research to understand not just the extent of poor mental health but how best to mitigate it within a high-demand occupational environment.

Substance misuse is reported as a coping mechanism for injury and occupational stress, with alcohol and drug use varying across studies (Davies et al., 2023; King et al., 2021; McConn-Palfreyman et al., 2019; Schefstad & Tiegel, 1997). Specifically, Schefstad and Tiegel (1997) study highlighted the stresses faced by stable staff such as having few transferrable skills, isolation, and minimal education. Although this study provided the first insight into alcohol and drug usage, the survey was designed for evaluating pre- and post-alcohol treatment effectiveness and not for determining the severity of the problem. Additionally, this study only focused on resident stable staff and not those who lived off-site. The combination of alcohol and drug use, minimal education, isolation, and limited transferable job skills, shows that the backstretch struggles with the occupational health of its employees. The establishment of on-site treatment facilities would significantly benefit this marginalized group of employees. The measures for alcohol and drug misuse in quantitative data can vary, however, most of the studies included did not disclose which scales or measures were used, which calls into question the reliability of the data and may explain the differing statistics reported. Further research is needed using validated questionnaires to gather data on alcohol and drug use among this population, allowing for comparison between different horse racing jurisdictions.

Sleep disturbances among trainers appear closely linked to occupational demands and broader organizational pressures (Bullock et al., 2019; Farmer et al., 2017). Poor sleep quality and increased psychological distress are likely influenced by long working hours, high job demands, and limited recovery time, reflecting systemic factors rather than individual causes alone. The studies also have methodological limitations, although both studies align in their findings, the inability to isolate key variables such as job role, workload, or specific stressors limits the conclusions that can be drawn. To build a clearer understanding of the relationship between sleep and mental health in the horse racing industry future research must gather detailed occupational categories and control for confounding variables such as working hours, role responsibilities, and employment conditions. More rigorous study design would help determine whether trainers are uniquely vulnerable or whether similar risks extend across other roles within the industry.

The industry also faces a staffing crisis, further compounding occupational health risks. High rates of presenteeism, turnover, and intentions to leave were reported (Bush et al., 2018; Butler et al., 2019; Filby et al., 2012; Juckes et al., 2021). Staffing shortages compromise horse welfare, reduce productivity, and increase the physical and emotional burden on the remaining staff (Butler et al., 2019; Filby et al., 2012). Personal and job-related pressures compel staff to attend work despite illness (Bush et al., 2018), while poor communication, inadequate training, and limited career progression contribute to workforce instability (Juckes et al., 2021). While these studies collectively underline critical factors that contribute to the staffing crisis, methodological limitations and differing participant work roles restrict generalizability of the findings. Nevertheless, recurrent themes emerged across the studies of poor communication, lack of recognition, and the emotional and physical toll of working in the horse racing industry, yet the intersection of these psychosocial factors with turnover remains underexplored. Further research should prioritize larger representative samples to determine the staffing shortage and the direct impact on staff well-being and horse welfare. To address the staffing crisis targeted interventions informed by these insights should be implemented, including professional development in workplace communication, establishing career development pathways, and delivering relevant training that enhances job satisfaction and retention.

Across multiple geographical contexts, the literature reveals a workforce that is under considerable strain, with physical injuries, poor mental health, low sleep quality, and substance misuse interrelated to systemic issues such as long hours, financial instability, and insubstantial support. These challenges are further exacerbated by staffing shortages and a workplace culture that discourages help-seeking and reinforces stigma. While individual studies have shed light on specific issues, the field lacks cohesive, critical exploration of how these factors interact over time. An integrated approach is needed to understand how the occupational health and well-being of stable staff and trainers is shaped by industry practices and cultural norms.

Future research should employ longitudinal and intervention-based designs, use validated assessment tools, and explore context-specific determinants of occupational health risks. Investigating the role of human, structural, and industry factors will be critical for developing effective strategies to reduce injury, improve mental health and sleep outcomes, mitigate substance misuse, and enhance workforce sustainability. Addressing these challenges requires integrated interventions targeting workplace culture, organizational practices, and industry-level policies to support the well-being, resilience, and retention of stable staff and trainers.

**Summary List**

- Stable staff and trainers face high rates of injuries, with musculoskeletal and horse-related trauma frequently underreported due to workplace cultures that normalize harm and discourage formal reporting.
- Mental health challenges are common, exacerbated by long hours, low pay, job insecurity, bullying, harassment, and limited access to support, particularly among migrant or minority staff.
- Substance misuse and poor sleep often co-occur with injury and stress, highlighting the interconnected nature of occupational risks in this workforce.
- Workforce shortages, poor training, and limited career progression contribute to staffing shortages, affecting both staff well-being and horse welfare, and indicate a need for systemic workplace interventions.
